# Decreasing utilization of tibial osteotomy and regional disparities: A population‐based study of all surgeries in Sweden from 2008 to 2023

**DOI:** 10.1002/jeo2.70386

**Published:** 2025-07-27

**Authors:** Gustav Nilsson, Viktor Schmidt, Michael Axenhus

**Affiliations:** ^1^ Department of Orthopaedic Surgery Danderyd University Hospital Stockholm Sweden; ^2^ Division of Orthopaedics, Department of Clinical Sciences at Danderyd Hospital Karolinska Institutet Stockholm Sweden

**Keywords:** population‐based study, Sweden, tibial osteotomy, trends

## Abstract

**Purpose:**

Tibial osteotomy (TO) is a surgical procedure used to treat unicompartmental gonarthrosis and correct lower extremity deformities. While historically effective, TO utilization has declined, possibly due to advances in alternative procedures such as unicompartmental knee arthroplasty (UKA) and total knee arthroplasty (TKA). The aim of this study is to describe and highlight demographic characteristics, trends in utilization and regional variance of TO in Sweden during 2008 to 2023 as well as estimate the usage by 2030.

**Methods:**

A retrospective cohort study was conducted using the Swedish National Patient Register, including all TO procedures performed between 1 January 2008 and 31 December 2023. Incidence rates per 100,000 inhabitants were calculated. Statistical analyses included descriptive statistics, Student's *t* tests for group comparisons, and regression modelling for future incidence trends.

**Results:**

A total of 4729 TO procedures were performed, averaging 296 per year. The overall incidence declined from 5.0 per 100,000 in 2008 to 2.3 in 2023. The decline was greater among men than women, narrowing the sex‐based incidence gap. Most TOs were performed in patients aged 45–54 years (35%), followed by 55–64 years (24%). Large regional variations were observed, with regions both over‐ and underperforming the national average of TOs significantly. Predictive modelling suggests a continued decline, with an estimated incidence of 0.8 per 100,000 by 2030.

**Conclusions:**

TO utilization in Sweden has declined and is estimated to continue decreasing in the future, likely due to increased UKA and TKA adoption. Regional disparities suggest variations in clinical practice and healthcare accessibility. Further research is needed to evaluate indications for TO as well as to establish the role of TO in current knee osteoarthritis management.

**Level of Evidence:**

Level II.

**Abbreviations**
ICD‐10: International Statistical Classification of Diseases and Related Health Problems version 10
NOMESCO: Nordic Medico Statistical Committee
NPR: Swedish National Patient Register
OA: osteoarthritis
SNBHW: Swedish National Board of Health and Welfare's National Patient Register
TKA: total knee arthroplasty
TO: tibial osteotomy
UKA: unicompartmental knee arthroplasty

## INTRODUCTION

Tibial osteotomy (TO) is a well‐documented surgical intervention striving to realign the knee joint. Common indications for this procedure are to treat unicompartmental osteoarthritis (OA) or correct lower extremity deformities, which may arise from fracture malunion, congenital deformities or other structural abnormalities [14, 16, 21, 24].

Utilized for the appropriate patient TO can be a successful treatment to delay or prevent the need for knee arthroplasty [3, 10]. The ideal patient is middle‐aged (40–60 years) and moderately physically active with a body mass index <30, isolated medial OA and no ligamentous instability. TO is contraindicated in patients with severe OA (Ahlback Grade ≥ III), multicompartmental OA, major malalignment and knee ROM < 120° [3, 22]. Advancements in surgical techniques, osteosynthesis material and patient selection have improved the results of TO over time. However, TO is associated with adverse events in addition to the potential need for later conversion to knee arthroplasty. Conversion arthroplasty may be associated with higher rates of postoperative infections and less range of motion than primary arthroplasty [17, 23].

Several studies have reported decreasing trends of TO [11, 12]. In Sweden, limited large‐scale data regarding trends in TO are available. No data have been published on the topic since the period 1998 to 2007, during which the number of high TO decreased by one third [27].

The aim of this study is to investigate the unknown landscape of TO from 2008 to 2023 in Sweden using large‐scale data from the Swedish National Patient Register (NPR). Specifically, the study seeks to describe and highlight demographic characteristics, trends in utilization, and regional disparities as well as conduct a predictive analysis.

## METHODS

### Study design

This study is a retrospective cohort study based on data from the NPR from 2008 to 2023. This study adhered to the RECORD guidelines [1].

### Setting

Swedish citizens are entitled to subsidised healthcare through the Swedish National Health Service, enabling widespread, high‐quality healthcare access. Swedish citizens are assigned a unique personal identification number that is utilized in interactions with healthcare providers and national healthcare registers, such as the NPR.

### Data source

The NPR is a nationwide register in Sweden administered by the Swedish National Board of Health and Welfare's National Patient Register (SNBHW) [25]. The NPR utilizes the International Statistical Classification of Diseases and Related Health Problems version 10 (ICD‐10) and Nordic Medico Statistical Committee (NOMESCO) coding system for categorization of surgical intervention, NOMESCO Classification of Surgical Procedures (NCSP) [19, 28]. All healthcare providers are required to report data to the NPR; thus, all orthopaedic departments (*n* = 54) in Sweden are engaged in the NPR. The NPR is validated for use in epidemiological and retrospective studies [13].

### Patients

Inclusion criteria:

1. Individuals with residency in Sweden at the time of surgery.
2. Individuals who underwent TO as specified by NOMESCO‐coding NGK59 between 1 January 2008 and 31 December 2023 in Sweden.

Exclusion criteria:

1. Age < 15 years.

### Variables

Sex was categorized as male or female. Age was stratified using 10‐year categories. Healthcare regions were categorized according to the official subdivision.

### Statistics

Analyses were performed using GraphPad Prism (version 10.3.1) and SPSS (version 29.1). Descriptive statistics were utilized and presented in whole numbers and percentages, excluding incidence data that were reported with one decimal. The incidence data were reported per 100,000 inhabitants and year. To determine significant differences between populations, Student's *t* tests were utilized, with a *p* value of ≤0.05 considered significant. 95% confidence intervals (CIs) were used where applicable. Regression analysis was conducted for predictions regarding future trends, considering linear, exponential and polynomial regression models to find the best fit for each incidence trend.

### Ethics

The study was performed using open‐source data and was therefore not subject to ethical review as per Swedish law.

## RESULTS

### Descriptive data and patients

**Figure 1:**
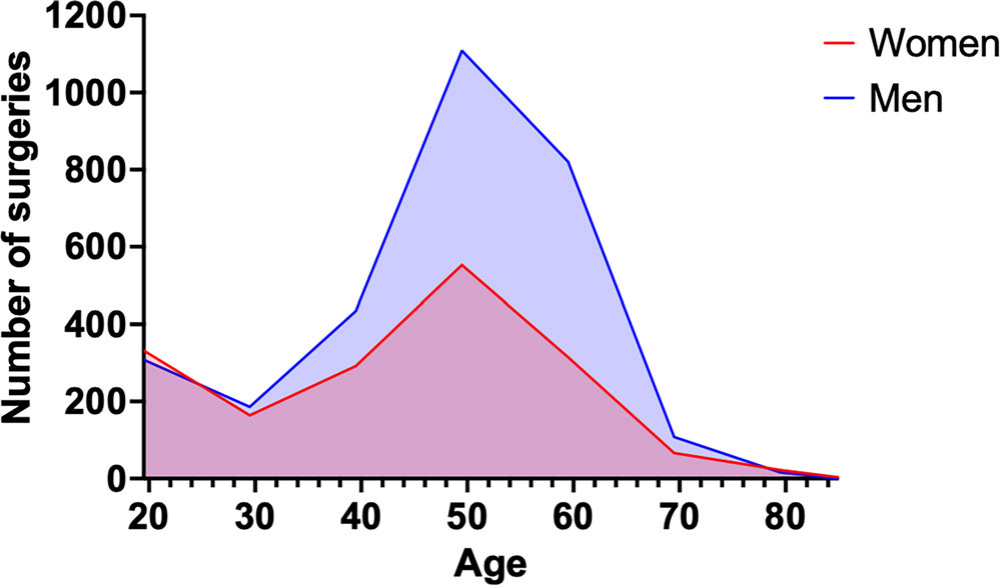
Age and sex distribution of tibial osteotomy in absolute numbers in Sweden (2008–2023).

During the 16‐year study period, a total of 4729 patients underwent TO, with an average of 296 surgeries per year. The number of surgeries decreased by 48% in total, ranging from 383 in 2008 to 200 in 2023. The majority were men (*n* = 2982), accounting for 63%. TO decreased by 58% among men and 25% among women during the study period. The majority of surgeries were performed in the age group 45–54 years (*n* = 1662), followed by 55–64 years (*n* = 1136), corresponding to 35% and 24%, respectively. For the age group ≥65 years, only 215 surgeries were performed between 2008 and 2023, corresponding to 5% of the total (Table 1). Up to the second decade of life, the number of surgeries was symmetrically distributed between men and women. After the age of 30, men were overrepresented. Both sexes displayed a bimodal distribution with a major peak in the fourth decade of life (Figure 1).

**Table 1:** Number of patients undergoing tibial osteotomy in Sweden (2008–2023).

	2008	2009	2010	2011	2012	2013	2014	2015	2016	2017	2018	2019	2020	2021	2022	2023	Total
Total	383	398	346	316	355	342	372	292	311	269	274	286	172	207	206	200	4729
Sex																	0
Men	261	255	231	207	238	226	237	168	189	163	166	190	103	122	117	109	2982
Women	122	143	115	109	117	116	135	124	122	106	108	96	69	85	89	91	1747
Age																	0
15–24	40	49	39	36	37	32	43	50	48	41	39	38	27	41	34	46	640
25–34	25	23	12	13	15	16	28	18	21	18	25	37	22	20	31	26	350
35–44	69	73	65	47	41	46	58	53	50	42	34	39	22	22	34	31	726
45–54	134	133	110	113	150	138	142	86	120	92	93	94	55	66	74	62	1662
55–64	102	104	108	83	100	92	83	73	58	59	72	59	42	49	26	26	1136
65–74	12	11	10	20	9	14	16	9	12	14	9	17	2	5	6	8	174
75–84	1	5	2	4	3	4	2	3	2	3	2	2	2	2	1	0	38
85+	0	0	0	0	0	0	0	0	0	0	0	0	0	2	0	1	3

**Figure 2:**
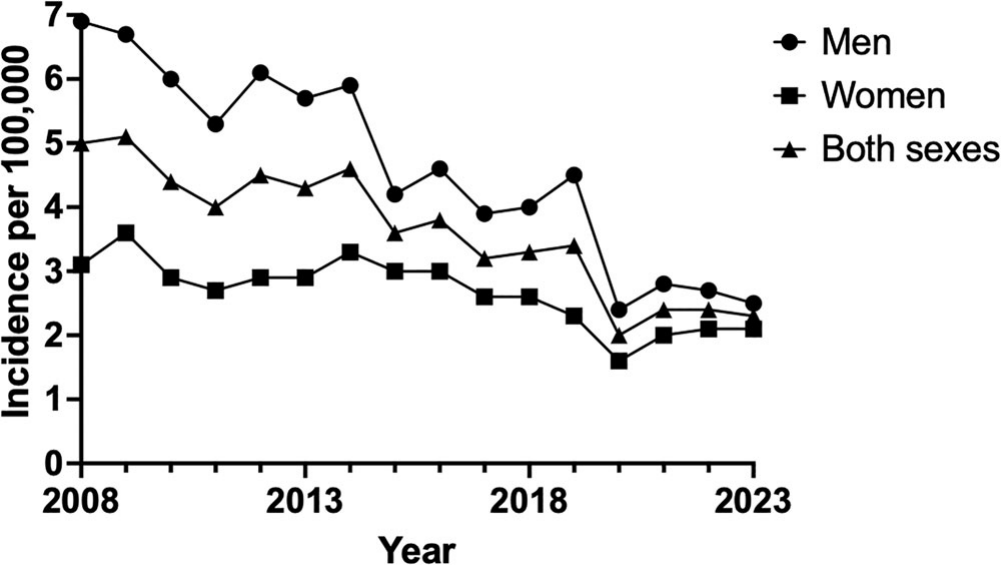
Incidence per 100,000 inhabitants undergoing tibial osteotomy in Sweden (2008–2023).

The incidence (per 100,000/year) of TO decreased by 54%, from 5.0 in 2008 to 2.3 in 2023. The decrease was seen for both sexes but was more prominent for men than women (64% and 32% respectively), resulting in a narrowing of the incidence sex gap. Looking at specific age groups, all displayed a clear trend of decreasing incidence, with the exception of those aged 15–24 and ≥85. The age group 15–24 displayed a pendulous trend with years of both increased and decreased incidence, while those aged ≥85 only underwent three TO in total, not enabling trend analysis (Tables 2 and 3, Figure 2).

**Table 2:** Incidence per 100,000 inhabitants undergoing tibial osteotomy in Sweden (2008–2023).

	2008	2009	2010	2011	2012	2013	2014	2015	2016	2017	2018	2019	2020	2021	2022	2023	Change 2008 to 2023
Total	5.0	5.1	4.4	4.0	4.5	4.3	4.6	3.6	3.8	3.2	3.3	3.4	2.0	2.4	2.4	2.3	−54%
Sex																	
Men	6.9	6.7	6.0	5.3	6.1	5.7	5.9	4.2	4.6	3.9	4.0	4.5	2.4	2.8	2.7	2.5	−64%
Women	3.1	3.6	2.9	2.7	2.9	2.9	3.3	3.0	3.0	2.6	2.6	2.3	1.6	2.0	2.1	2.1	−32%
Age																	
15–24	3.3	4.0	3.1	2.9	3.0	2.6	3.6	4.2	4.1	3.5	3.4	3.3	2.3	3.5	2.9	3.8	15%
25–34	2.2	2.0	1.0	1.1	1.3	1.3	2.2	1.4	1.6	1.3	1.8	2.6	1.5	1.4	2.1	1.8	−18%
35–44	5.3	5.6	5.0	3.7	3.2	3.7	4.6	4.2	4.0	3.3	2.7	3.0	1.7	1.7	2.6	2.3	−57%
45–54	11.4	11.1	9.0	9.1	11.9	10.8	11.0	6.6	9.1	6.9	6.9	7.0	4.1	5.0	5.6	4.7	−59%
55–64	8.4	8.6	9.1	7.0	8.6	8.0	7.3	6.4	5.1	5.1	6.2	5.0	3.5	4.0	2.1	2.1	−75%
65–74	1.4	1.3	1.1	2.1	0.9	1.4	1.5	0.8	1.1	1.3	0.8	1.5	0.2	0.5	0.6	0.8	−43%
75–84	0.2	0.9	0.4	0.7	0.5	0.7	0.3	0.5	0.3	0.5	0.3	0.3	0.3	0.3	0.1	0.0	−100%
85+	0.0	0.0	0.0	0.0	0.0	0.0	0.0	0.0	0.0	0.0	0.0	0.0	0.0	0.8	0.0	0.4	N/A

**Table 3:** Regional incidence of tibial osteotomy in Sweden (2008–2023).

	2008	2009	2010	2011	2012	2013	2014	2015	2016	2017	2018	2019	2020	2021	2022	2023	Change 2008–2009 to 2022–2023
Gävleborg	7.7	9.8	9	8.1	11.1	10.3	10.2	8.9	8.9	11.3	13	17.1	3.8	6.7	7.1	5.4	−29%
Kalmar	9.6	7.6	12.1	5	6.5	11.1	7.5	4.5	6.4	6.4	7.4	5.4	4.4	1	2.4	2.4	−72%
Kronoberg	10.6	19	11.1	7.8	6.5	9	4.5	5.7	7.6	4.4	4.3	1.2	0.6	1.2	0.6	0	−98%
Jönköping	6.1	10.1	7.2	4.6	6.1	5.3	5.7	6.3	3.5	3.8	4.4	4.7	2.4	3.7	3.7	4.3	−51%
Östergötland	6.2	4.2	8.1	5	3.9	5	9.6	8.7	6.5	4	4	3.1	2.3	5.9	1.8	1.8	−65%
Uppsala	2.6	2.9	2.9	3.6	6	3.2	6.6	5.9	8.8	7.3	7.5	3.5	6	6.5	2.7	2.7	−2%
Skåne	10.1	8.4	6.3	5.5	5.6	5.7	6.3	3.2	4.9	3.8	2.4	3.2	2.2	1.3	2.3	1.7	−78%
Gotland	2.1	4.1	4.1	4.1	8.2	6.2	0	4.1	4.1	6.1	6	11.9	0	3.9	1.9	5.8	24%
Halland	3.3	3.3	4.9	4.4	6	4.4	8.3	3.9	1.5	6.4	4.5	5.2	4	2.5	2.1	1.8	−41%
Västra Götaland	5.9	4.4	4	3.7	4.9	4	3.6	3.5	4.7	3.7	4.1	4.7	3.2	3.3	3	2.6	−46%
Västerbotten	5.1	6.9	5	5	5.5	5.5	6.4	2.7	1.8	4.5	2.2	2.2	1.8	1.3	2.6	1.7	−64%
All regions	5	5.1	4.4	4	4.5	4.3	4.6	3.6	3.8	3.2	3.3	3.4	2	2.4	2.4	2.3	−53%
Västmanland	2.4	1.9	2.8	6.1	6.5	7	3.2	3.7	1.4	1.3	1.8	3.5	1.3	4.8	2.2	2.2	2%
Dalarna	5.6	6	3.8	3.4	3	3	3	0.9	3	2.1	3.8	2.5	0.4	2.1	3.3	2.1	−53%
Södermanland	0.9	4.9	2.7	3.5	3.1	2.6	3.9	3	2.1	1.7	1.7	3.3	0.4	2	3.2	2.8	3%
Västernorrland	4.4	5.4	3.4	4.4	2.5	2.9	1	1.5	2.9	3.4	2	0.5	0.5	0	0.5	1	−85%
Jämtland	3.7	1.9	4.7	2.8	0.9	5.6	2.8	4.7	2.8	1.9	0	1.8	0.9	1.8	0	0	−100%
Stockholm	2	2.7	2	2.9	2.8	2.5	3.3	2.2	2.4	1.4	2	1.9	0.9	1.4	2.3	2.7	6%
Örebro	3	2.1	3	2.1	3.4	3.8	3.8	3.7	2.1	0	0.8	1.6	0.4	0.8	1.6	0.4	−61%
Blekinge	3.9	3.1	1.5	3.1	2.3	0.8	0.8	4.6	0.8	0.8	3	0	2.3	0	0	2.3	−67%
Norrbotten	2.8	2.8	1.9	0.5	1.9	1.9	2.8	2.4	0.9	0	0.5	0.5	0	1.4	0.5	1.4	−66%
Värmland	1.3	3.9	1.7	1.7	3	1.7	1.7	0.9	0.4	1.3	0.4	0.4	1.7	0.4	0	1.3	−75%

### Regional analysis

The incidence (per 100,000/year) of TO varies between regions in Sweden. Furthermore, for several regions, major interannual variations were observed. Comparing the combined incidence of 2008–2009 with 2022–2023, the variance ranged from −100% (Jämtland) to +24% (Gotland) and was for all regions combined −53%. (Table 3).

**Figure 3:**
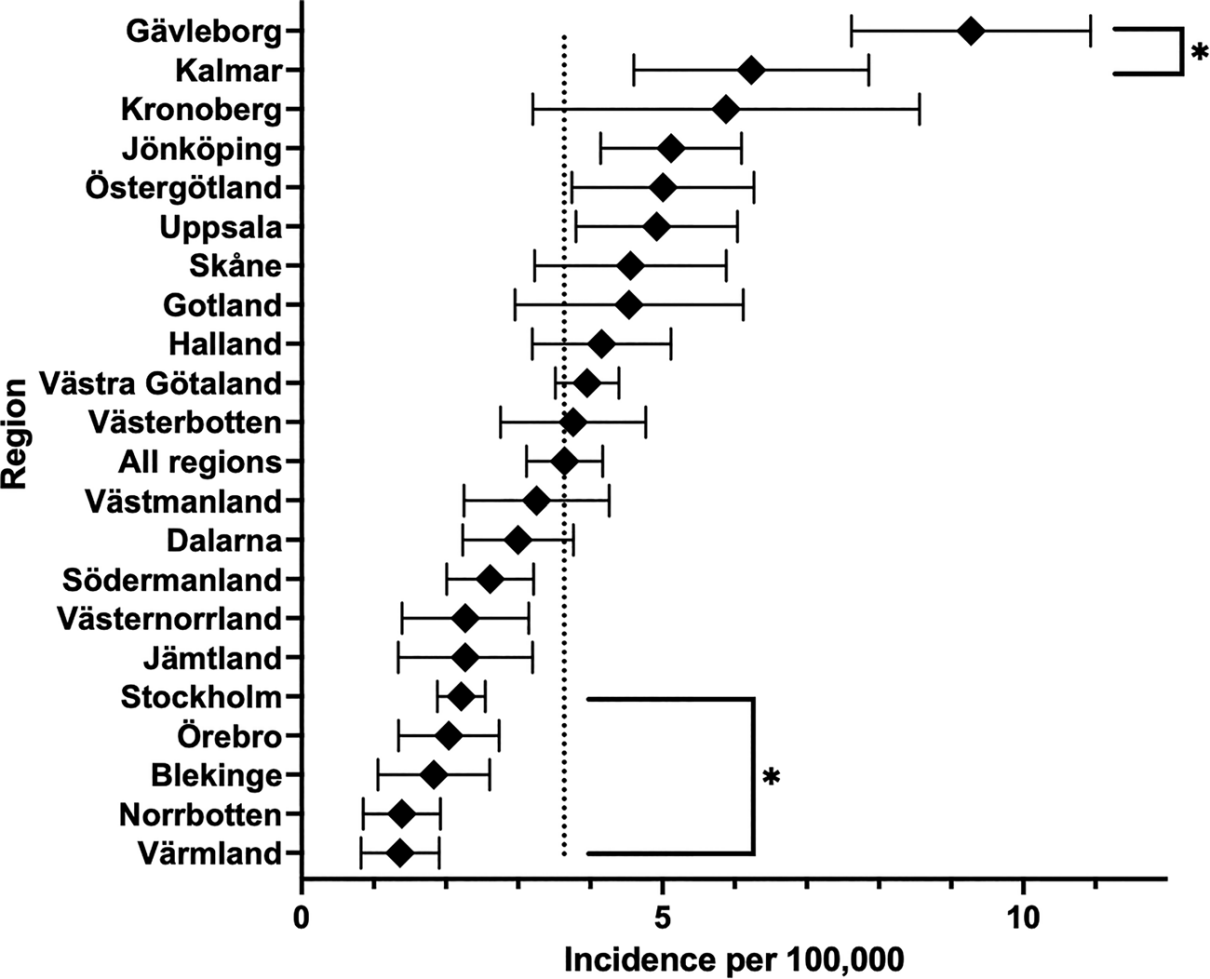
Mean regional incidence of tibial osteotomy (2008–2023) with 95% confidence intervals. *Comparative analysis shows a significant difference between the national average and regions with *p* < 0.05.

The national mean incidence (per 100,00/year) of TO between 2008 and 2023 was 3.6. Two regions performed significantly more TO than the national average, Kalmar and Gävleborg, with a mean incidence of 6.2 and 9.3, respectively. On the other side of the spectrum, five regions performed significantly less TO than the national average, with Norrbotten and Värmland performing the least, both with a mean incidence of 1.4 (Figure 3).

### Predictive analysis

**Figure 4:**
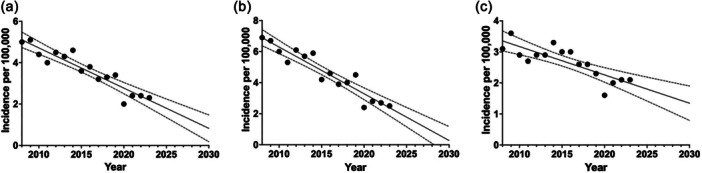
Predictive analysis of mean incidence of tibial osteotomy in Sweden with 95% confidence intervals (2008–2023), (a) all, (b) men and (c) women.

Predictive analysis of mean incidence (per 100,000/year) of TO indicates a decreasing trend for both sexes combined. The decrease is primarily driven by a larger decline among men than women. By 2030, the incidence is predicted to drop to 0.8 (CI: 0.1–1.3) for both sexes combined, 0.3 (CI: −0.6 to 1.0) for men and 1.3 (CI: 0.9–1.9) for women (Figure 4).

## DISCUSSION

Our study presents a comprehensive analysis of TO trends in Sweden from 2008 to 2023, revealing a significant decline in utilization, demographic shifts, and regional variations. Over the 16‐year period, the total number of TO procedures decreased by 48%, with an overall incidence reduction of 54%, from 5.0 per 100,000 inhabitants in 2008 to 2.3 in 2023, and is estimated to decrease henceforth. The decline in TO was more pronounced among men compared to women, resulting in a narrowing of the incidence gap between the sexes. The majority of procedures were performed in patients aged 45–54 years, with a bimodal distribution peaking in the fourth and fifth decades of life. Regional disparities were evident, with some regions, such as Kalmar and Gävleborg, performing significantly more procedures than the national average, while others, including Norrbotten and Värmland, performed fewer.

These findings align with international and national reports showing a decreasing trend in TO utilization [11, 12, 27]. This is likely due to a shift favouring unicompartmental knee arthroplasty (UKA) and total knee arthroplasty (TKA) over TO [6, 17, 23]. Advantages and disadvantages of primary knee arthroplasty versus TO have been extensively studied. TO for OA have a 10‐year survival exceeding 80%, often delaying the need for TKA by many years. However, conversion from TO to TKA is reported to be more technically challenging and may be associated with poorer long‐term outcomes in some studies [15, 23]. Adverse events such as decreased range of motion and higher infection rates have been observed compared to primary TKA, while other studies have reported similar long‐term results [2, 9, 18, 26]. The potential risks associated with conversion from TO to TKA, alongside the advancements in knee arthroplasty, may in part account for the decreased use of TO globally, despite the improved functionality of TO‐related implants such as locking plates. Furthermore, demographic trends such as increasing obesity and decreased physical activity could favour TKA over TO [4, 7, 8]. For comparison, data from the NPR regarding femoral osteotomy show a rather stable incidence around 1.0 per 100,000 people for both men and women from 2008 to 2023. However, we are not able to identify the level of osteotomy or association with TO. In certain situations, TO and FO are combined, but often FO is performed as a separate procedure for valgus malalignment, precluding any conclusions.

The regional variability in TO incidence across Sweden highlights discrepancies in clinical practice. While some regions, such as Kalmar and Gävleborg, performed significantly more TOs than the national average, others, such as Norrbotten and Värmland, recorded significantly lower rates. These differences may stem from regional variations in healthcare access, surgeon expertise, referral patterns or institutional preferences. No in‐depth analysis regarding the specific regional demographics in relation to their incidence has been conducted. However, no clear trend is evident. For example, the two largest healthcare regions with similar demographics, Stockholm and Västra Götaland, displayed a mean incidence difference of almost 100%, while Stockholm and Jämtland displayed more similarity, although the latter is a rural area with disparate demographics. Similar disparities have been reported in Italy and Korea, where regional trends indicate variability in surgical decision‐making [11, 12]. Identifying and understanding these regional differences is crucial for the analysis of healthcare accessibility and equality, enabling equitable access to joint‐preserving procedures.

Predictive modelling suggests a continued decline in TO incidence, projecting an overall rate of 0.8 per 100,000 inhabitants by 2030. This trend is primarily driven by decreasing TO utilization among men. The increasing adoption of UKA and TKA as preferred treatments for knee OA has likely contributed to this shift, as evidenced by global trends demonstrating a steady rise in arthroplasty rates at the expense of TO [5, 20].

While this study provides valuable insights, several limitations must be acknowledged. The inability to differentiate between the specific location of TO and/or the diagnosis warranting surgery in this dataset limits analysis. This lack of detailed indication‐specific data prevents a more nuanced interpretation of the underlying reasons in the observed decline in TO, such as shifting indications, demographic changes or surgeon preferences, as well as analysis of trends in subgroups, such as those undergoing TO for OA versus post‐traumatic or congenital deformities. This limitation highlights a need for more detailed, prospective data to further evaluate these findings, given that differences in our dataset are inferred and not directly measured. Conducting predictive analysis based on observed data using regression models has several well‐known shortcomings, such as over‐ and underfitting, extrapolation risk and the inability to adjust for external factors. This study's strengths include having a nationwide, population‐based design with comprehensive data from the NPR, which boasts high completeness and reliability. The large sample size enables relatively accurate predictive modelling.

In conclusion, TO utilization in Sweden has significantly declined over the past 16 years, mirroring international trends. Men undergo TO more often than women, but the sex gap has narrowed. Regional disparities in TO rates highlight a large variability in clinical practice across Sweden. Predictive analysis suggests a continued downward trajectory urging further research to establish the reasons behind this trend.

## AUTHOR CONTRIBUTIONS

**Michael Axenhus**: Conceptualization; methodology; software; validation; formal analysis; investigation; resources; data curation; writing—review and editing; visualization; supervision. **Viktor Schmidt**: Conceptualization; methodology; validation; data curation; writing—review and editing. **Gustav Nilsson**: Software, formal analysis, investigation, writing—original draft, visualization.

## CONFLICT OF INTEREST STATEMENT

The authors declare no conflicts of interest.

## ETHICS STATEMENT

The ethics statement is not available.

## Data Availability

The data used in this study were obtained from the website of the SNBHW and are publicly available for anyone to download and use. The data sets can be obtained from the NPR directly (https://www.socialstyrelsen.se/en/statistics-and-data/statistics/statistical-databases/).
